# Analysis of risk factors for positive surgical margin after laparoscopic radical prostatectomy with and without neoadjuvant hormonal therapy

**DOI:** 10.3389/fendo.2023.1270594

**Published:** 2023-10-24

**Authors:** Fangming Wang, Gang Zhang, Yuzhe Tang, Yunpeng Wang, Jianxing Li, Nianzeng Xing

**Affiliations:** ^1^ Department of Urology, Tsinghua University Affiliated Beijing Tsinghua Changgung Hospital, Tsinghua University Clinical Institute, Beijing, China; ^2^ Department of Outpatient, The Second Medical Center & National Clinical Research Center for Geriatric Diseases, Chinese PLA General Hospital, Beijing, China; ^3^ Department of Urology, National Cancer Center/National Clinical Research Center for Cancer/Cancer Hospital, Chinese Academy of Medical Sciences and Peking Union Medical College, Beijing, China

**Keywords:** neoadjuvant hormonal therapy, positive surgical margin, laparoscopic radical prostatectomy, risk factors, prostate cancer

## Abstract

**Background:**

Positive surgical margins (PSM) is not only an independent risk factor for recurrence, metastasis, and prognosis, but also an important indicator of adjuvant therapy for prostate cancer (PCa) patients treated with radical prostatectomy (RP). At present, there are few reports analyzing risk factors of PSM in laparoscopic RP (LRP), especially for those PCa cases who accepted neoadjuvant hormonal therapy (NHT). Hence, the aim of the current study was to explore risk factors for PSM after LRP in PCa patients with and without NHT.

**Methods:**

The clinicopathological data of patients who underwent LRP from January 2012 to July 2020 was retrospectively analyzed. Risk factors for PSM after LRP in NHT and non-NHT groups were respectively explored.

**Results:**

The overall PSM rate was 33.3% (90/270), PSM rate was 39.3% (64/163) in patients without NHT and 24.3% (26/107) in those with NHT. The apex was the most common location of PSM in non-NHT group (68.8%, 44/64), while the fundus was the most common location of PSM in NHT group (57.7%, 15/26). Multiple logistic regression revealed that body mass index (BMI), PSA, ISUP grade after LRP, pathological stage T (pT) and pathological lymph node status (pN) were independent factors affecting the PSM for patients without NHT (OR=1.160, 95%CI:1.034-1.301, p=0.011; OR=3.385, 95%CI:1.386-8.268, p=0.007; OR=3.541, 95%CI:1.008-12.444, p=0.049; OR=4.577, 95%CI:2.163-9.686, p<0.001; OR=3.572, 95%CI:1.124-11.347, p=0.031), while pT, pN, and lymphovascular invasion (LVI) were independent risk factors affecting PSM for patients with NHT (OR=18.434, 95%CI:4.976-68.297, p<0.001; OR=7.181, 95%CI:2.089-24.689, p=0.002; OR=3.545, 95%CI:1.109-11.327, p=0.033).

**Conclusions:**

The apex was the most common location in NHT group, and BMI, PSA, ISUP after LRP, pT and pN were independent risk factors affecting PSM for NHT patients; while the fundus was the most common location in non-NHT group, and pT, pN, and LVI were independent risk factors affecting PSM for non-NHT patients.

## Introduction

Prostate cancer (PCa) is the second most common cancer and the fifth most common cause of cancer death among men worldwide in 2020 (1). Although there are emerging diagnostic biomarkers for the detection of PCa (2, 3), PCa is usually diagnosed by prostate biopsy prompted by a blood test to measure prostate-specific antigen (PSA) levels and/or digital rectal examination. Localized PCa can be cured by radical prostatectomy (RP) or radiotherapy. PCa relapses after RP are treated with salvage radiotherapy and/or androgen deprivation therapy (ADT) for local relapse, or with ADT combined with chemotherapy for systemic relapse. Advanced PCa often progresses despite ADT and is then considered castration-resistant and incurable. Patients with localized disease at a low to intermediate risk of recurrence generally have a favorable outcome of 99% overall survival for 10 years if the disease is treated at an early stage (4). As mentioned, RP is the most common treatment options for localized PCa, and increasingly used as an important step for the treatment of advanced local, and even early metastatic cases when indicated (5). Postoperative histopathology report of RP specimens including pathological stage T (pT), lymph node status (pN), grade, perineural invasion (PNI), lymphovascular invasion (LVI), and positive surgical margin (PSM) is of crucial importance to clarify the stage, make decisions for adjuvant treatment, and predict patient outcome after RP (6, 7). A PSM is defined as cancer at the inked margin of the specimen. This can follow from incising into the extraprostatic cancer in patients with extracapsular extension or by incision into an otherwise organ-confined cancer (8). Although controversial, most studies validated that PSM is an independent risk factor for biochemical recurrence, local recurrence, distant metastasis, and prognosis (9–11), and exerts stressful impact on patients and family members (12). Moreover, PSM is an important indicator for postoperative adjuvant hormonal therapy or radiotherapy after RP (13, 14). Therefore, urologists should be familiar with factors influencing PSM and spare no effort to avoid the occurrence of PSM during operation. At present, studies reporting risk factors of PSM mainly focused on robot-assisted LRP and open RP, and there are few reports analyzing risk factors of PSM in conventional LRP. Besides, almost all studies exploring risk factors of PSM excluded PCa cases which have accepted neoadjuvant hormonal therapy (NHT). Until now, it remains largely unknown which clinicopathological parameters could be risk factors for PSM in PCa patients treated with NHT prior to RP. Hence, the aim of the current study was to describe the locations of PSM and the clinicopathological characteristics including BMI, PSA, grade, stage, PNI, and LVI and identify the independent risk factors affecting PSM of RP specimen in the Chinese PCa patients who underwent conventional LRP with and without NHT, respectively. Our results could have clinical implications for the prediction of PSM and guidance of individual LRP surgery, postoperative adjuvant therapy, and evaluation of prognosis.

## Materials and methods

### General information

A total of 275 patients with PCa who underwent extraperitoneal LRP and pelvic lymph node dissection performed by the same surgeon in Tsinghua University Affiliated Beijing Tsinghua Changgung Hospital and Cancer Hospital and Chinese Academy of Medical Sciences and Peking Union Medical College between January 2012 and October 2020 were reviewed. Inclusion criteria of the study were pathologically confirmed prostate adenocarcinoma in LRP specimen. Exclusion criteria of the study were (1) medical history of receiving any radiotherapy or chemotherapy before LRP; (2) LRP converted to open operation. For the present study, we withdrew 2 patients who underwent radiotherapy and 3 cases whose LRP procedures converted into open surgical procedures, and collected 270 PCa cases who met the recruitment standard and were identified for analysis. Mean age was 67.4 ± 6.7 years, mean body mass index (BMI) was 25.4 ± 3.2 kg/m^2^. Median preoperative PSA was 15.3 (9.0-28.7) ng/ml, median preoperative prostate volume was 35.2 (23.0-46.2) ml. All demographic and clinicopathological data including age, BMI, hypertension, diabetes mellitus, history of pelvic surgery, preoperative PSA, prostate volume, the interval between biopsy and RP, NHT treatment, and postoperative pathological results including pT and pN, ISUP (international society of urological pathology) grade, PSM and exact locations, PNI, and LVI were collected from the hospital information system.

### Definitions

All LRP samples were routinely sent to the pathological department for diagnosis, and were fixed, serially sectioned and processed according to the well-established protocols. Pathologic factors analyzed included grade and tumor stage, PSM, PNI, and LVI. The cancer grade assessment was performed according to the ISUP 2014 classification system (15). The pT was evaluated on the basis of a prostatectomy specimen according to the American Joint Committee on Cancer (AJCC) TNM classification of malignant tumors in 2017 (AJCC, pT2-T4, Nx,0,1) (16). pT1+pT2 were categorized as localized PCa and pT3+pT4 were categorized as locally advanced PCa. PSM was defined as tumor extending to the inked surface of the prostatectomy specimen that the surgeon has cut across (17). The apex, fundus, body, and vas deferens or seminal vesicles are four locations of PSM in the pathological report. PNI is defined as cancer tracking along or around a nerve within the perineural space (18). PNI was defined as the invasion of cancer cells in, around, and through the nerves. Diagnostic criterion for LVI was defined as the presence of tumor cells within an endothelial-lined space that is usually devoid of a muscular wall(www.cap.org/protocols-and-guidelines/cancerreporting-tools/cancer-protocol templates). The pathology of the presence of PSM, PNI, and LVI was reviewed by 2 senior pathologists through comprehensive analysis of H&E staining results. For controversial cases, a third pathologist was invited to reach group agreement.

### Statistical analysis

Data were expressed as means ± SD or median with interquartile range for continuous variables and number (percentage) for categorical variables. The differences between continuous variables were analyzed by unpaired t-tests or MannWhitney U tests as appropriate. Categorical variables were analyzed by χ2-test. At first, all PCa subjects were divided into two groups according to whether receiving NHT or not: non-NHT and NHT groups, and clinicopathological variables including PSM were compared between the two groups. Then all cases were divided into two groups according to PSM status: non-PSM and PSM groups, and clinicopathological variables were compared between the two groups. Significant clinicopathological risk factors for PSM examined by univariate logistic regression analysis were further analyzed by multivariate logistic regression analysis to determine the independent risk factors of PSM in non-NHT and NHT groups, respectively. All tests were two-sided and a p-value <0.05 was considered significant. The statistical analyses were performed with SPSS version 22.0 software (Chicago, IL, USA).

## Results

### Characteristics of non-NHT and NHT patients

The clinicopathological characteristics of the enrolled PCa subjects were shown in **Table 1**.The mean age was 67.4 ± 6.7 years, mean BMI was 25.4 ± 3.2 kg/m^2^. Median preoperative PSA was 15.3 (9.0-28.7) ng/ml, median preoperative prostate volume was 35.2 (23.0-46.2) ml. The overall PSM rate was 33.3% (90/270), and the accumulated distribution of locations was as following: apex (62.2%, 56/90), fundus (41.1%, 37/90), body (37.8%, 34/90), vas deferens/seminal vesicles (7.8%, 7/90).

**Table 1:** Baseline characteristics of the PCa subjects underwent LRP according to the NHT stratification.

	All subjects (n=270)	Non-NHT (n=163)	NHT (n=107)	p value
Demographic characteristics				
Age (years)	67.4 ± 6.7	66.7 ± 6.8	68.4 ± 6.4	**0.032**
BMI (kg/m^2^)	25.4 ± 3.2	25.0 ± 3.2	26.0 ± 3.2	**0.017**
Hypertension (n (%))	116 (43.0%)	72 (44.2%)	44 (41.1%)	0.706
Diabetes mellitus (n (%))	55 (20.6%)	33 (20.5%)	22 (20.8%)	0.959
Pelvic surgery history (n (%))	20 (7.4%)	13 (8.0%)	7 (6.5%)	0.813
Clinicopathological parameters				
PSA (ng/ml)	15.3 (9.0-28.7)	11.6 (7.7-21.2)	23.4 (12.3-45.6)	**<0.001**
ISUP grade of biopsy (n (%))				**<0.001**
1	58 (22.2)	40 (25.6)	18 (17.1)	
2	61 (23.4)	46 (29.5)	15 (14.3)	
3	57 (21.8)	36 (23.1)	21 (20.0)	
4	46 (17.6)	23 (14.7)	23 (21.9)	
5	39 (14.9)	11 (7.1)	28 (26.7)	
Without biopsy	9 (3.3)	7 (4.3)	2 (1.9)	
Prostate volume (ml)	35.2 (23, 46.2)	36.7 (28.2,46.9)	29.8 (21.4,45.8)	**0.041**
Interval time from puncture to LRP (days)	51.0 (36.0-105.5)	42.0 (32.0-55.3)	100.0 (52.0-178.0)	**<0.001**
ISUP after LRP (n (%))				**<0.001**
1	35 (13.0)	26 (16.0)	9 (8.4)	
2	69 (25.6)	52 (31.9)	17 (15.9)	
3	72 (26.7)	48 (29.4)	24 (22.4)	
4	22 (8.1)	10 (6.1)	12 (11.2)	
5	72 (26.7)	27 (16.6)	45 (42.1)	
Pathological stage (n (%))				0.767
T1+T2	156 (57.8)	93 (57.1)	63 (58.9)	
T3+T4	114 (42.2)	70 (42.9)	44 (41.1)	
N (n (%))				**0.036**
N0	234 (86.7)	147 (90.2)	87 (81.3)	
N1	36 (13.3)	16 (9.8)	20 (18.7)	
M (n (%))				**0.003**
M0	254 (94.1)	159 (97.5)	95 (88.8)	
M1	16 (5.9)	4 (2.5)	12 (11.2)	
PSM (n (%))	90 (33.3)	64 (39.3%)	26 (24.3)	**0.011**
PSM locations (n (%))				0.123
apex	28 (31.1)	24 (37.5)	4 (15.4)	
apex+fundus	11 (12.2)	7 (10.9)	4 (15.4)	
apex+body	8 (8.9)	8 (12.5)	0	
apex+fundus+body	5 (5.6)	3 (4.7)	2 (7.7)	
apex+fundus+vas deferens/seminal vesicles	4 (4.4)	2 (3.1)	2 (7.7)	
fundus	11 (12.2)	6 (9.4)	5 (19.2)	
fundus+body	5 (5.6)	4 (6.3)	1 (3.8)	
fundus+body+vas deferens	1 (1.1)	0	1 (3.8)	
body	15 (16.7)	9 (14.1)	6 (23.1)	
vas deferens/ seminal vesicles	2 (2.2)	1 (1.6)	1 (3.8)	
Accumulated PSM locations (n (%))				**0.048**
apex	56 (62.2)	44 (68.8)	12 (46.2)	
fundus	37 (41.1)	22 (34.4)	15 (57.7)	
body	34 (37.8)	24 (37.5)	10 (38.5)	
vas deferens/ seminal vesicles	7 (7.8)	3 (4.7)	4 (15.4)	
PNI (n (%))	180 (66.7)	105 (64.4)	75 (70.1)	0.333
LVI (n (%))	37 (13.7)	18 (11.0)	19 (17.8)	0.117

Data are expressed as n (%), mean ± SD, or median (interquartile range). The bold value indicated statistical significance. PCa, prostate cancer; LRP, laparoscopic radical prostatectomy; NHT, neoadjuvant hormonal therapy; BMI, body mass index; PSA, prostate-specific antigen; ISUP, International Society of Urological Pathology; M, metastasis; PSM, positive surgical margin; PNI, perineural invasion; LVI, lymphovascular invasion.

Among all 270 PCa patients, 163 did not receive NHT and 107 received NHT. Among NHT patients, 82 cases accepted maximal androgen blockade (MAB) therapy with median course of 3 months; 3 cases accepted simple castration therapy with median course of 3 months; 22 cases accepted anti-androgen monotherapy (AAM) with median course of 1 month. The PSM rate of non-NHT and NHT group was 39.3% (64/163), 24.3% (26/107), respectively, and the difference was significantly different (p=0.011). The apex was the most common location of PSM in non-NHT group (68.8%, 44/64), while the fundus was the most common location of PSM in NHT group (57.7%, 15/26). There were significant statistical difference in terms of age, BMI, PSA, ISUP grade of biopsy and after LRP, prostate volume, the interval between biopsy and RP, pN status, and metastasis between non-NHT and NHT groups (p=0.032, p=0.017, p<0.001, p<0.001, 0.041, p<0.001, p=0.036, p=0.003, respectively), while there were no significant difference regarding pT, PNI, and LVI between the two groups (p=0.767, 0.333, 0.117, respectively).

### Relation of PSM with the clinicopathological characteristics of PCa

As shown in **Table 2**, the percentages of locally advanced PCa (pT3+pT4), pN (+), cases without NHT and cases with LVI were significantly higher in PSM group (n=90), when compared with non-PSM group (n=180) (p<0.001, p=0.004, p=0.012, p=0.015).

**Table 2:** Relation of PSM with the clinicopathological characteristics of PCa patients underwent LRP.

	Non-PSM (N=180)	PSM (N=90)	p value
Age (years)	67.0 ± 6.6	68.2 ± 6.8	0.180
BMI (kg/m^2^)	25.3 ± 3.2	25.7 ± 3.3	0.292
Hypertension (n (%))	78 (67.2)	38 (32.8)	0.897
Diabetes mellitus (n (%))	38 (69.1)	17 (30.9)	0.872
Pelvic surgery history (n (%))	12 (60.0)	8 (40.0)	0.623
PSA (ng/ml)	14.0 (8.5-27.3)	18.1 (9.5-30.1)	0.068
PSA group (n (%))			0.181
<10ng/ml	55 (73.3)	20 (26.7)	
10-20ng/ml	50 (68.5)	23 (31.5)	
>20ng/ml	59 (60.2)	39 (39.8)	
Missing (n)	16	8	
ISUP grade of biopsy (n (%))			0.219
1	41 (70.7)	17 (29.3)	
2	46 (75.4)	15 (24.6)	
3	37 (64.9)	20 (35.1)	
4	29 (63.0)	17 (37.0)	
5	21 (53.8)	18 (46.2)	
Without biopsy	6	3	
Prostate volume (ml)	38.1 ± 19.9	39.0 ± 19.2	0.722
Interval time from puncture to LRP (days)	51.0 (36.0-117.0)	50.5 (35.5-79.3)	0.284
ISUP after LRP (n (%))			0.205
1	26 (74.3)	9 (25.7)	
2	49 (71.0)	20 (29.0)	
3	49 (68.1)	23 (31.9)	
4	16 (72.7)	6 (27.3)	
5	40 (55.6)	32 (44.4)	
Pathological stage (n (%))			**<0.001**
T1+T2	128 (82.1)	28 (17.9)	
T3+T4	52 (45.6)	62 (54.4)	
N (n (%))			**0.004**
N0	164 (70.1)	70 (29.9)	
N1	16 (44.4)	20 (55.6)	
M (n (%))			0.786
M0	170 (66.9)	84 (33.1)	
M1	10 (62.5)	6 (37.5)	
NHT (n (%))			**0.012**
Yes	81 (75.7)	26 (24.3)	
No	99 (60.7)	64 (39.3)	
PNI (n (%))			0.494
Yes	117 (65.0)	63 (35.0)	
No	63 (70.0)	27 (30.0)	
LVI (n (%))			**0.015**
Yes	18 (48.6)	19 (51.4)	
No	162 (69.5)	71 (30.5)	

Data are expressed as n (%), mean ± SD, or median (interquartile range). The bold value indicated statistical significance. PSM, positive surgical margin; PCa, prostate cancer; LRP, laparoscopic radical prostatectomy; BMI, body mass index; PSA, prostate-specific antigen;

ISUP, International Society of Urological Pathology; M, metastasis; NHT, neoadjuvant hormonal therapy; PNI, perineural invasion; LVI, lymphovascular invasion.

There were no significant difference in terms of age, BMI, hypertension, diabetes, history of pelvic surgery, PSA before biopsy, ISUP grade before biopsy and after LRP, prostate volume, the interval between biopsy and RP, metastasis, and PNI between non-PSM and PSM groups (all p>0.05).

### Correlation of clinicopathological variables with the presence of PSM in PCa patients without NHT

We performed univariate and multivariate logistic regression analysis to evaluate the correlations of clinicopathological variables with PSM in non-NHT group. As shown in **Table 3**, BMI, PSA before biopsy, ISUP grade after LRP, pT, and pN were found to be significantly and positively correlated with the presence of PSM in univariate logistic regression analysis (p<0.05). In the stepwise multivariate regression analysis, we added and adjusted confounding factors including age, BMI, hypertension, diabetes, history of pelvic surgery, prostate volume, and ultimately revealed that the correlations of BMI, PSA before biopsy, ISUP grade after LRP, pT, and pN with PSM remained significant after adjustment for confounding factors (OR=1.160, 95%CI:1.034-1.301, p=0.011; OR=3.385, 95%CI:1.386-8.268, p=0.007;OR=3.541, 95%CI:1.008-12.444, p=0.049; OR=4.577, 95%CI:2.163-9.686, p<0.001; OR=3.572, 95%CI:1.124-11.347, p=0.031, respectively).

**Table 3:** Logistic regression analysis for correlation of clinicopathological variables with the presence of PSM in PCa patients without NHT.

	univariate mode	p value	Multivariate mode	p value
	OR (95%CI)		OR (95%CI)	
Age	1.026 (0.978-1.076)	0.292		
BMI	1.112 (1.003-1.233)	**0.044**	1.160 (1.034-1.301)	**0.011**
Hypertension	0.876 (0.464-1.652)	0.682		
Diabetes	1.228 (0.564-2.673)	0.605		
History of pelvic surgery	0.667 (0.196-2.263)	0.516		
PSA before puncture	1.033 (1.006-1.061)	**0.017**	1.031 (1.003-1.059)	**0.028**
<10ng/ml	Reference		Reference	
10-20ng/ml	1.720 (0.751-3.940)	0.200	1.401 (0.572-3.432)	0.460
>20ng/ml	3.663 (1.563-8.587)	**0.003**	3.385 (1.386-8.268)	**0.007**
ISUP grade of biopsy				
1	Reference			
2	0.657 (0.265-1.625)	0.363		
3	1.491 (0.597-3.725)	0.392		
4	1.282 (0.451-3.641)	0.641		
5	2.000 (0.519-7.703)	0.314		
preoperative prostate volume	1.008 (0.991-1.025)	0.350		
Interval time from puncture to LRP (days)	1.002 (0.998-1.006)	0.380		
ISUP after LRP				
1	Reference		Reference	
2	1.093 (0.396-3.014)	0.864	0.966 (0.317-2.946)	0.952
3	1.750 (0.638-4.802)	0.277	1.762 (0.558-5.563)	0.334
4	0.563 (0.097-3.267)	0.521	0.236 (0.021-2.648)	0.242
5	3.273 (1.054-10.158)	**0.040**	3.541 (1.008-12.444)	**0.049**
postoperative pathological stage T	3.833 (1.975-7.440)	**<0.001**	4.577 (2.163-9.686)	**<0.001**
pathological lymph node involvement	3.902 (1.287-11.833)	**0.016**	3.572 (1.124-11.347)	**0.031**
M	4.820 (0.490-47.387)	0.177		
PNI	1.222 (0.630-2.369)	0.533		
LVI	2.728 (0.997-7.460)	0.051	2.803 (0.961-8.175)	0.059

Univariate and multivariate logistic regression analysis were performed. The multivariate model was adjusted for age, BMI, hypertension, diabetes, history of pelvic surgery, prostate volume. The bold value indicated statistical significance. The dependent variable was PSM of PCa. PSM, positive surgical margin; PCa, prostate cancer; NHT, neoadjuvant hormonal therapy; BMI, body mass index; PSA, prostate-specific antigen; ISUP, International Society of Urological Pathology; LRP, laparoscopic radical prostatectomy; M, metastasis; PNI, perineural invasion; LVI, lymphovascular invasion.

### Correlation of clinicopathological variables with the presence of PSM in PCa patients with NHT

As shown in **Table 4**, ISUP grade of biopsy, pT, pN, and LVI were found to be significantly and positively correlated with the presence of PSM (p<0.05) in PCa patients with NHT in univariate logistic regression analysis. In the stepwise multivariate regression analysis, only pT, pN, and LVI remained significantly correlated with the presence of PSM after adjusting the mentioned confounding factors in NHT group (OR=18.434, 95%CI: 4.976-68.297, p<0.001; OR=7.181, 95%CI: 2.089-24.689, p=0.002; OR=3.545, 95%CI:1.109-11.327, p=0.033).

**Table 4:** Logistic regression analysis for correlation of clinicopathological variables with the presence of PSM in PCa patients with NHT.

	univariate mode	p value	Multivariate mode	p value
	OR (95%CI)		OR (95%CI)	
Age	1.057 (0.981-1.137)	0.144		
BMI	0.974 (0.846-1.121)	0.713		
Hypertension	1.067 (0.436-2.612)	0.888		
Diabetes	0.445 (0.120-1.651)	0.226		
History of pelvic surgery	4.727 (0.984-22.721)	0.052		
PSA before puncture	1.003 (0.993-1.012)	0.599		
<10ng/ml	Reference			
10-20ng/ml	0.600 (0.143-2.511)	0.484		
>20ng/ml	0.937 (0.284-3.087)	0.914		
ISUP grade of biopsy				
1	Reference		Reference	
2	1.231 (0.152-9.972)	0.846	1.205 (0.133-10.909)	0.868
3	1.333 (0.197-9.020)	0.768	1.288 (0.173-9.609)	0.805
4	3.500 (0.628-19.496)	0.153	3.627 (0.573-22.950)	0.171
5	6.000 (1.153-31.228)	**0.033**	5.718 (0.997-32.801)	0.050
preoperative prostate volume	0.988 (0.965-1.013)	0.346		
Interval time from puncture to LRP (days)	1.000 (1.000-1.001)	0.347		
ISUP after LRP				
1	Reference			
2	1.714 (0.152-19.359)	0.663		
3	0.727 (0.058-9.159)	0.805		
4	4.000 (0.363-44.113)	0.258		
5	4.414 (0.506-38.526)	0.179		
postoperative pathological stage T	14.75 (4.567-47.642)	**<0.001**	18.434 (4.976-68.297)	**<0.001**
pathological lymph node involvement	3.369 (1.205-9.418)	**0.021**	7.181 (2.089-24.689)	**0.002**
M	1.043 (0.260-4.183)	0.952		
PNI	1.576 (0.566-4.390)	0.384		
LVI	2.828 (0.992-8.064)	**0.052**	3.545 (1.109-11.327)	**0.033**

Univariate and multivariate logistic regression analysis were performed. The multivariate model was adjusted for age, BMI, hypertension, diabetes, history of pelvic surgery, prostate volume. The bold value indicated statistical significance. The dependent variable was PSM of PCa. PSM, positive surgical margin; PCa, prostate cancer; NHT, neoadjuvant hormonal therapy; BMI, body mass index; PSA, prostate-specific antigen; ISUP, International Society of Urological Pathology; LRP, laparoscopic radical prostatectomy; M, metastasis; PNI, perineural invasion; LVI, lymphovascular invasion.

## Discussion

LRP is the most common treatment option for localized and locally advanced PCa in China. The desirable outcomes after RP are to achieve “trifecta” including oncological outcomes, continence, and potency (19). Afterwards, some urologists presented the new concept of “pentafecta” outcomes, which includes complications and PSM on top of the traditional trifecta outcomes (20). No matter “trifecta” or “pentafecta” standard is adopted, controlling oncological outcomes is the most important task of LRP. However, PSM occurred in postoperative pathological result, which would influence the controlling of oncological outcomes and affect the prognosis unfavorably. Therefore, knowing the common location and risk factors of PSM is crucial and has great clinical value for developing the surgical strategy and guiding adjuvant therapy after LRP. Our current study firstly demonstrated the locations of PSM and investigated the correlation between clinicopathological parameters and PSM in PCa patients who underwent LRP with and without NHT, and demonstrated that BMI, PSA, ISUP grade after LRP, pT and pN were independent risk factors affecting PSM for patients without NHT, while pT, pN, and LVI were independent risk factors affecting PSM for patients with NHT. Our results have clinical implications for the prediction of PSM and guidance of operation especially for those receiving NHT.

At present, there were many studies reporting PSM rate globally. As summarized in **Table 5** (21–29), PSM rate exceeded 25% in Chinese PCa patients, while PSM range was approximately 12-16.3% in the counterpart of other countries, the difference of which can be explained by the more advanced PCa percentages in China than other countries. The overall PSM rate was 33.3%, and PSM without NHT was 39.3% in our study, which were a little higher than those reported in some other centers in China (26–28). Tumor location within the prostate can influence the risk of PSM. In the current study, there were four locations of PSM according to the pathological report: apex, fundus, body, and vas deferens/seminal vesicles. Of the 90 cases of PSM, the apex accounted the largest proportion, the only and accumulated proportion of apex were 31.1% and 62.2%, respectively. Our results were in consistent with one study reporting that the apex accounted for 59.1% among all PSM locations after LRP (21). We summarized three reasons for the high PSM of apex: (a) The apex is deep and closely neighbored with deep dorsal vein complex, erectile nerve, and rectum, and thus relatively difficult to get exposed during operation; (b) The apex lacks of barrier of capsule, resulting in more susceptible to tumor invasion; (c) There are many variations in the shape of the urethral of apex, cutting perpendicular to urethral may increase the risks of tumor residue. Tumors located at the ventral or dorsal part of the prostate may also have different influence on PSM rate, which needs further study.

**Table 5:** Summary of literature reporting PSM rate and related independent risk factors for PSM in PCa patients underwent LRP.

Literature	Total cases (n)	PSM rate	Independent risk factors
Secin FP (21), 2007	407	12%	PSA, Gleason score, low volume, interfascial pathway
Sooriakumaran P (22), 2014	4918	16.3%	/
Yao Zhu (23), 2015	94	17%	pT
Wei Song (24), 2015	61	19.7%	TNM stage after puncture, PSA, positive percentage of the needle biopsy.
Louie-Johnsun MW (25), 2016	2943	15.9%(T2:9.8%;T3a:30.8%;T3b:39.2%)	/
Rong Yang (26), 2017	296	29.1%	Gleason score, PNI, number of positive cores in the biopsy specimen, and low prostate volume.
Zheng Zhang (27), 2019	177	32.2%	pT
Xiaojun Tian (28), 2019	418	34%	the percentage of positive cores in preoperative biopsy, clinical stage, f/t PSA, and age.
Pingxin Zhang (29), 2020	99	26.3%	pT
**The current study, 2023**	270	33.3%	
**Without NHT**	163	39.3%	BMI, PSA before needle biopsy, postoperative ISUP grade, pT and pN
**With NHT**	107	24.3%	pT, pN, and LVI

PSM, positive surgical margin; PCa, prostate cancer; LRP, laparoscopic radical prostatectomy; PSA, prostate-specific antigen; pT, pathological stage T; PNI, perineural invasion; f/t,free/total; NHT, neoadjuvant hormonal therapy; BMI, body mass index; ISUP, International Society of Urological Pathology; pN, pathological lymph node involvement; LVI, lymphovascular invasion. /, not reported.

The risk factors for PSM reported by different centers were different (**Table 5**), and the subjects of almost all these studies analyzed were the PCa cases without NHT. As the advanced PCa accounts higher in China than the developed countries, the percentage of PCa patients receiving NHT may increase with the promotion and application of NHT. The protocol of NHT includes MAB, castration, and AAM, lasting 3-9 months before surgery. In our study, 39.6% of cases received NHT. Among the 107 patients with NHT, 82 cases accepted MAB therapy with median course of 3 months; 3 cases accepted castration therapy with median course of 3 months; 22 cases accepted AAM with median course of 1 month. The protocol and duration of NHT were influenced by many factors such as doctor’s suggestion, economic status of the family, and psychological demands of the patient.

Our study demonstrated that BMI, PSA, ISUP grade after LRP, pT and pN were independent risk factors affecting PSM for patients without NHT. Of note, BMI can reflect the infiltration of pelvic fat. For obese patients, pelvic fat can increase the difficulty of separation due to adhesion and increase the risk of PSM. However, the influence was relatively little with only OR value of 1.16. Previously conducted studies did not find BMI was an independent risk factor for PSM (21, 23, 24, 26–29). Therefore, the influence of BMI on PSM may be negligible. In addition to BMI, certain patient factors including history of pelvic surgery, and anatomical variations can impact the risk of PSM. However, in our study, we find that the correlation of pelvic surgery history with PSM was insignificant after adjustment for confounding factors, which could be explained by the small sample of cases with previous surgeries in the pelvic area. Secin FP, et al. (21) reported that preoperative PSA was an independent risk factor for PSM (OR=1.15, 1.04-1.28,p<0.01). Jason P. Izard, et al. (30) also reported that PSA was an independent risk factor for PSM, and the PSM rate of PCa patients with PSA<4.0, 4-9.9, and>10 ng/ml was 12%, 20%, and 28%, respectively. In line with these reports, our analysis showed that the OR of PSM for those with PSA>20ng/ml was 3.385. PSA was secreted by prostatic epithelial cells, and PSA elevation indicates that the highly aggressive PCa cells damage the tissue barrier, increasing the risk of PSM. Our result demonstrated that the OR of postoperative ISUP grade 5 for PSM was 3.54, which was consistent with the conclusions reached by Secin FP (21), Yang Rong (26), and Jason P. Izard (30). It is noteworthy that ISUP grade after LRP is generally higher than the biopsy ISUP grade for the same case, and could more objectively reflect the tumor grade. In our study, pT was an independent factor for PSM in non-NHT patients with the highest OR value among all risk factors (OR=4.577), which was partly in consistent with the studies (23, 27, 29) reporting pT was the only independent factor for PSM in PCa patients without NHT. We divided pT into localized (T1+T2) and locally advanced (T3+T4) stage, as advanced PCa break through the capsule or invade the neighbor tissues, therefore the PSM rate was higher in T3+T4 groups. Besides, we revealed that pN was also an independent risk factor for PSM in non-NHT patients, we believed that local lymph node involvement indicate the high aggressiveness of local tumor. It is challenging for surgeons to completely remove large and aggressive tumors while preserving negative surgical margins. Collectively, compared with previous studies (21–30), there exist differences and similarities regarding the risk factors for PSM in PCa cases without NHT in our study, which could be explained by the sample size, the surgeon experience and technique, and other factors.

Next, let’s focus on those PCa patients with NHT. Naiki, et al. (31) reported that PSM rate was 27.8% (20/72) in PCa patients with NHT, which was consistent with our result demonstrating PSM rate was 24.3% in NHT groups. At present, it is generally recognized that NHT can reduce prostate volumes and stages, decrease PSA levels and PSM rate, but can’t improve the long-term survival. The seemingly contradictory conclusion could be explained as follows: NHT could improve the local control of tumor, but could not eradicate the micrometastases (32). However, from another perspective, as NHT can reduce PSM rate, and PSM rate was closely correlated with biochemical recurrence and prognosis, NHT is inferred to improve prognosis. Therefore, the exact relationships among NHT, PSM and prognosis need further exploration.

In the current study, compared with non-NHT patients, the NHT counterparts were older and with higher BMI and ISUP grade before biopsy. In other words, the baseline parameters of the two groups were significantly different, which was the reason why we did not observe the decreasing effect of NHT on postoperative stage. Our research firstly demonstrated that pT, pN and LVI were independent risk factors for PSM in patients treated with NHT before LRP. Generally speaking, the frequency of pT, pN, and LVI would decrease after NHT, however, if one of these parameters was still positive after NHT, then the local invasion was supposed to be severe and increase the PSM rate.

It is noteworthy that the fundus rather than apex was the most common location for NHT patients, which was different from non-NHT patients. The could be explained as follows: on one hand, NHT reduced prostate volume, and thus made apex exposed more clearly during operation, helping to avoid PSM of apex; on the other hand, NHT could cause evident adhesion between prostate fundus and bladder neck, making the boundary difficult to distinguish and causing PSM. The cases in our study were all performed in extraperitoneal approach, and thus eliminated the bias caused by different surgery approaches.

Some studies reported that low prostate volume was an independent risk factor for PSM (21, 26), which was not observed in our study. Some researcher (27) believed that the smaller the prostate is, the larger the ratio between the tumor and the prostate, and the easier the tumor exceed the limited range of pathological staging and cause PSM. On the other hand, the smaller the prostate is, the wider the operation space, reducing the difficulty of surgery and risk of PSM. Therefore, we considered that the combined effect of the two factors is the reason why the low prostate volume was not the independent factor of PSM in our study. Moreover, the technique of surgeons was closely related to PSM, all LRP were performed by the same surgeon, who has proficient laparoscopic operation skills. The degree of neurovascular bundle (NVB) dissection was determined by the operating surgeon on the basis of the plane where the dissection took place. There are three degrees of NVB dissection: intrafascial (complete NVB sparing), interfascial (partial NVB sparing), and extrafascial (complete/almost complete NVB resection). Generally, in the current study, the surgeon preferred to spare NVB for the localized low to intermediate-risk cases, and do not spare NVB for the cases with extracapsular extension. In this way, we maximally reduced the bias caused by operation skills in our study.

The present study might have two potential clinical implications. First, urologists should dissect the urethral of the prostatic apex more carefully when performing LRP on PCa patients without NHT, and tackle the bladder neck more carefully when performing LRP on cases with NHT, respectively, to avoid PSM incidence as much as possible; second, preoperative BMI, PSA, biopsy grade, and stage could be used to predict the PSM of postoperative specimen and to estimate the prognosis, and the influencing factors should be considered by laparoscopic surgeons when planning the operation to decrease the incidence of PSMs.

## Conclusions

In conclusion, our study first demonstrated that PSM rate was 24.3% and the fundus was the most common location of PSM in Chinese patients with NHT. The pT, pN, and LVI were independent factors affecting the PSM for Chinese patients with NHT. By comparison, PSM rate was 39.3% and the apex was the most common location of PSM in Chinese patients without NHT. BMI, PSA before needle biopsy, postoperative ISUP grade, pT and pN were independent factors affecting the PSM for patients without NHT. Our results provide reliable clinical implications for guidance of LRP and prediction of PSM especially for those PCa patients receiving NHT. For example, surgeons should pay more attention to the resection extent when tackling the boundary of prostate fundus and bladder neck in PCa patients with NHT, and use preoperative clinicopathological parameters to predict the PSM risks. The limitation of our study was that there might exist selection bias because the data analyzed in the study were derived from a single center, and studies from different hospitals are needed for validation of our results.

## Data availability statement

The raw data supporting the conclusions of this article will be made available by the authors, without undue reservation.

## Ethics statement

The studies involving humans were approved by Ethical committee of National Cancer Center. The studies were conducted in accordance with the local legislation and institutional requirements. The participants provided their written informed consent to participate in this study. Written informed consent was obtained from the individual(s) for the publication of any potentially identifiable images or data included in this article.

## Author contributions

FW: Conceptualization, Data curation, Formal Analysis, Funding acquisition, Investigation, Methodology, Software, Validation, Visualization, Writing – original draft, Writing – review & editing. GZ: Writing – review & editing, Methodology, Validation, Investigation, Resources, Software. YT: Writing – review & editing, Methodology, Formal analysis, Resources, Software. YW: Investigation, Writing – original draft, Writing – review & editing. JL: Conceptualization, Formal Analysis, Methodology, Project administration, Supervision, Writing – review & editing. NX: Conceptualization, Funding acquisition, Project administration, Resources, Supervision, Writing – review & editing.
